# The Nuclear Legacy in Appalachia

**DOI:** 10.13023/jah.0201.07

**Published:** 2020-01-26

**Authors:** Michele Morrone, Harold Perkins

**Affiliations:** Ohio University; Ohio University

**Keywords:** Appalachia, Cold War, rural health, enriched uranium, nuclear weapons, nuclear fuel, radiation, pollution

## Abstract

Nestled in the rolling hills of Appalachia Ohio is a reminder of the role that the region played in winning the Cold War. For more than 40 years in rural Pike County, the 3,700-acre Portsmouth Gaseous Diffusion Plant (PORTS), or the “A-Plant” as the locals refer to it, enriched uranium for use in nuclear weapons. While the facility produced nuclear fuel for national security, it simultaneously exposed plant workers to chemicals and radiation and discharged pollution into the surrounding community. The A-Plant is now being demolished and the site repurposed. However, the site continues to affect the community as, for example, a middle school near it was closed in late spring of 2019 due to alarming levels of radiation detected in the building.

Nestled in the rolling hills of Appalachia Ohio is a reminder of the role that the region played in winning the Cold War. For more than 40 years in rural Pike County, the 3,700-acre Portsmouth Gaseous Diffusion Plant (PORTS), or the “A-Plant” as the locals refer to it, enriched uranium for use in nuclear weapons. While the facility produced nuclear fuel for national security, it simultaneously exposed plant workers to chemicals and radiation and discharged pollution into the surrounding community. The A-Plant is now being demolished and the site repurposed. However, the site continues to affect the community as, for example, a middle school near it was closed in late spring of 2019 due to alarming levels of radiation detected in the building.^1^

This is yet one more public health challenge residents in this rural county endure. By several indicators, Appalachia Ohio is less healthy than the rest of the state and Pike County is historically one of the unhealthiest places in Ohio.^2^ Pike County is not unique in its social and behavioral challenges contributing to poor health across Appalachia. It is, however, different from other counties in Ohio because the legacy of PORTS creates unusual problems for people who worked in, and continue to live around, the facility. For example, *in addition* to radiation releases, during the site cleanup numerous organic chemicals were uncovered raising significant questions about long-term environmental health impacts. One of these chemicals, (TCE), is a solvent—and known human carcinogen—used to clean and degrease machinery at the facility.^3^ TCE is documented in groundwater plumes underneath and adjacent to PORTS and more than 37,000 pounds of TCE were removed from the site as part of its remediation.^4^

Recently, a group of former security guards at PORTS provided us a list of more than 70 colleagues suffering a range of illnesses: At least 12 were diagnosed with an unusual and aggressive form of prostate cancer prior to age 65. The guards believe their occupational exposures while protecting the A-Plant are the cause of these cancers. It is of course difficult to identify specific causes of prostate cancer, but the guards’ concerns warrant investigating occupational exposures to chemicals like TCE as potential risk factors.

The guards are perplexed and frustrated because their prostate cancers do not qualify them for federal benefits related to occupational illnesses associated with nuclear facilities. Eligibility for benefits depends on a causation, where it is “at least as likely as not” that exposure to a toxic substance was a significant factor in aggravating, contributing to, or causing the claimed illness. Interestingly, some former security guards do receive benefits for hearing loss related to TCE exposure. This makes investigating a potential link between TCE exposure and aggressive forms of prostate cancer that much more urgent for the guards as they see the federal government recognizing TCE as a causal factor in some forms of disease but not prostate cancer. Their hope is that it is a short step for officials to make a link between their TCE exposure and their cancer.

We interviewed some of the former security guards to hear their stories about what it was like to work at the A-Plant while it was enriching uranium. Some of the most compelling stories come from those men diagnosed with both prostate cancer and hearing loss. They know they were exposed to chemicals while they were working. As one of the guards we interviewed recalled:

“I know I was exposed to hazardous substances at the Atomic Plant…One of those substances, I’m trying to think of what it is called, that they used in the process that you could smell it once in a while, they used to release it, what is that called? And they had cleaning substances all over the place I understand that our gun cleaning stuff that we used, the oil and solvent, was not healthy for the hearing either, I don’t know. I couldn’t tell you all the chemicals, you know you go through these buildings and you could smell stuff, but you really don’t know what it is.”

Another guard provided a harrowing account of responding to chemical releases without any personal protective equipment:

“Me and (another guard) were in the PW (product withdrawal) area in the 326 building, that’s where the whole hot stuff of uranium is stored. So, we left to go to lunch… and we couldn’t breathe. I mean, no oxygen whatsoever. We took off running just about as hard as we could go, once we got 20 or 25 yards away, then we could breathe again. ...[maintenance workers] were changing out cold traps, they had masks on of course and whole uniforms. We were no further than about 15 or 20 yards away, they didn’t notify us that they were going to do this. We got to headquarters…and they said, ‘go to the hospital.’ So, we went over to the hospital… and both of our throats were raw and we both had fevers.”

The guards told us they had no idea if what they were exposed to was dangerous. One said:

“We used that stuff to clean motor parts and pump parts you know to cut grease off, we dump them in that tank and they come out spotless like brand new, it was good stuff for that, but I know it was very dangerous…as far as monitoring (for exposure) to trichlor, no way…”

Other former security guards recounted for us incidents of acute and long-term chemical exposures in the workplace. While they were uncertain about the chemicals they were exposed to, based on documented chemicals during the ongoing cleanup, it is “at least as likely as not” that at least some of their exposure was to TCE.

In summary, TCE is documented extensively at the A-Plant and the federal government acknowledges that hearing loss among some former employees is connected to their exposure to the chemical while working at the facility. Former security guards believe their unusual and aggressive prostate cancers are linked to TCE exposure too. Their concerns are not unfounded as previous studies suggest an association between TCE and prostate cancer.^5^–^9^ These aspects combine to raise questions about possible connections between TCE and prostate cancer in men who worked at PORTS.

The former guards’ stories humanize the effects of occupational exposures to chemicals like TCE and thus underscore the need for additional research examining the chemical’s potential connections to prostate cancer, among other diseases. The guards hope future research could lead to more programs for preventing, monitoring, and treating the disease. This is important because this is one of many environmental exposures unique to Appalachia where mortality rates from most cancers are already higher than in other regions. More specifically, however, further study of occupational exposure to TCE is important because these men believe they were serving their country while securing nuclear material at PORTS. They want the federal government to recognize their service by providing them care for their cancers.
